# In silico analysis reveals mir-98-5p as a potential inhibitor of tumor cell proliferation and metastasis in colorectal cancer by targeting the fzd3 receptor of the Wnt signaling pathway

**DOI:** 10.1186/s43141-023-00532-7

**Published:** 2023-07-19

**Authors:** Mutebi John Kenneth, Tushar Ahmed Shishir, Fahim Kabir Monjurul Haque

**Affiliations:** 1 Biotechnology Program, Department of Mathematics and Natural Sciences, BRAC University, Mohakhali, 1212 Dhaka, Bangladesh; 2 Microbiology Program, Department of Mathematics and Natural Sciences, BRAC University, Mohakhali, 1212 Dhaka, Bangladesh

**Keywords:** MicroRNAs, Colorectal cancer, WNT pathway, Frizzled receptors, miR-98-5p

## Abstract

**Background:**

Colorectal Cancer (CRC) is the third most common cancer type and the second leading cause of cancer-related deaths worldwide. However, the existing treatment, as well as prognosis strategies for CRC patients, need to be improved in order to increase the chance of survival. Targeted therapies of CRC, as opposed to ordinary therapies, target key biological features and pathways of cancerous cells hence minimizing the subsequent damage to normal cells. MicroRNAs have been reported to play a crucial role in inhibiting and/or suppressing major pathways in various cancer types by targeting transcripts of key genes in such pathways.

**Methods:**

The purpose of this study was to analyze in silico the differentially expressed genes from five microarray datasets of patients with CRC. Furthermore, miRNAs were investigated to inhibit cancer cell proliferation and metastasis by targeting a key gene—frizzled receptor 3 (FZD3) in the Wnt signaling pathway.

**Results:**

The Wnt pathway receptor FZD3 is upregulated in CRC along with other pathway genes, which play a critical role in tumorigenesis. In contrast, miR-98-5p inhibits the activity of FZD3 by binding directly to the 3′UTR of its mRNA, therefore exerting a suppressor effect on colorectal tumors.

**Conclusion:**

The study reveals miR-98-5p as a novel target of FZD3 and an inhibitor of the Wnt signaling pathway hence being a potential candidate for developing targeted therapies against CRC.

**Supplementary Information:**

The online version contains supplementary material available at 10.1186/s43141-023-00532-7.

## Background

In 2020, the World Health Organization (WHO) reported that cancer was one of the leading causes of death, claiming nearly ten million lives [1]. Among all types of cancer, colon and rectal cancer, also known as colorectal cancer (CRC), is the third most commonly diagnosed type worldwide, and it caused 935,000 deaths in the year 2020, making it the second deadliest type of cancer ahead of liver, stomach, and breast cancer [2]. As of now, there are three main treatment options for CRC: surgical resection, chemotherapy, immunotherapy, or any combination of these therapies. Nevertheless, the effectiveness varies from patient to patient, especially in cases of locally invasive or metastatic cancer. We must therefore understand how this disease progresses in order to identify biomarkers that can be used to identify potential therapeutic targets in order to improve the prognosis and treatment of CRC patients.

It is possible to improve treatment for CRC with targeted therapeutic agents that target unique biological features and pathways involved in tumor progression. In contrast to other therapeutics that kill both cancerous and normal cells, these work best in cancer treatment because they target the biological features of cancerous cells only [3]. Several pathways and processes in cancerous cells can be turned off by targeted therapy, including angiogenesis, proliferation, apoptosis inhibition, differentiation, the RAS pathway, the Wnt signaling pathway, the PI3K pathway, and the cell cycle pathway [4].

A number of studies shown that activation of the highly conserved Wnt pathway in a deviant manner is a driving factor in the tumorigenesis of most human cancers, with a strong emphasis on CRC [5]. This pathway controls β-catenin, a key modulator for signal transduction in CRC through phosphorylation and ubiquitin-mediated degradation. This regulation involves key scaffold proteins such as AXIN and disheveled (DVL) which disrupt the β-catenin destruction complex that is contains 3 core proteins; adenomatous polyposis coli (APC), glycogen synthase kinase 3 beta (GSK3β), and casein kinase 1 (CK1) [6]. When the destruction complex is disrupted, β-catenin will no longer be degraded hence leading to its accumulation as free β-catenin in the cytoplasm (Piawah and Venook, 2019b), which is a hallmark of CRC progression (Cheng et al. 2019) when it translocates to the nucleus [5]. This translocated beta-catenin in association with two major transcriptional factors, i.e., T cell factor (TCF) and lymphoid enhancer-binding factor (LEF), displaces their repressor molecule Groucho. The β-catenin/TCF/LEF complex, in association with other co-activators, forms a transcriptional complex that leads to the expression of target genes of Wnt, which include MYC, CCND1, AXIN2, Cyclin D1, among others [7, 8]. These target genes are mostly oncogenes [9]. Moreover, the abnormal up-regulation of the Wnt signaling pathway is facilitated by APC mutations, which is a negative regulator of this pathway [6]. These mutations mainly lead to the loss-of-function of APC hence upregulating the Wnt signaling pathway and facilitating CRC cell proliferation and enhanced anti-apoptosis abilities through overexpression of the target genes of this pathway [9].

Wnt signaling pathway is characterized as either canonical, which is β-catenin dependent or non-canonical, which is β-catenin independent. However, the initiation of signaling events in both pathways involves the binding of Wnt molecules to frizzled receptors and other related receptors like the Low-density lipoprotein Receptor-related Protein 5/6 (LRP5/6)/ROR2/RYK for signal transduction initiation [9]. Frizzled (FZD) is a family of 10 transmembrane proteins which serve as receptors of the Wnt pathway, with every FZD member having a favored Wnt ligand [10]. Various studies have indicated that excessive activation of the Wnt signaling pathway may be a result of a loss-of-function mutation in E3 ubiquitin ligases ring-finger protein 43 (RNF43), through ubiquitin-mediated degradation blockage of frizzled receptors and LRP5/6 co-receptors. Since this is a frequently detected phenomenon in CRC [8], it, therefore, indicates that signal transduction by the Wnt pathway can be influenced as levels of expression for key components of the pathway get altered [5]. Since Wnt is the most implicated pathway in CRC, disrupting the pathway signal transduction through downregulating the expression of crucial pathway components such as FZD receptors can be a therapeutic strategy for CRC.

Human frizzled homolog 3 protein (FZD3) is located on chromosome 8p21 and is expressed in skeletal muscles, pancreas, cerebellum, stomach, kidney, and among other tissues [11]. A number of studies have shown that FZD3 is up-regulated in tissues from lung squamous cell carcinomas, lymphomas, Ewing sarcomas, and myeloma, among other cancers [12, 13]. In their study, Wong and colleagues (2013) reported that FZD3 was 100% expressed in CRC spacemen, 89% in colorectal adenomas, and 75% in colorectal polyp spacemen [12]. Therefore, there is no doubt that FZD3 plays a critical role in the development and progression of CRC, making it a potential candidate for preventative interventions.

Recent studies have demonstrated that microRNAs (miRNAs) may be effective inhibitors of proliferation, growth, and metastasis of CRC cells by targeting FZD receptors and oncogenes [13–15]. These single-stranded noncoding RNAs bind to the 3′ untranslated region (3′ UTR) of the target gene mRNA, thereby negatively regulating its expression. This results in the cleavage of the target gene or repression of its translation, thereby inhibiting the production of the target protein [14]. The interaction of MicroRNAs with FZD mRNAs influences the expression of FZD proteins and the Wnt pathway as a result (Smith et al. 2021). Identifying miRNAs that inhibit the expression of FZD genes in different cancers is a reported therapeutic strategy for human cancer [10]. In comparison to other FZD receptors, the FZD3 receptor has received relatively little attention in human cancers, especially colorectal cancer. It is, therefore, the purpose of this study to identify a suitable miRNA target for FZD3 receptor mRNA and to demonstrate its effectiveness as an inhibitor. The finding of this study would allow the clinical evaluation of the potential of miRNA in inhibiting CRC progression and its consideration as a therapeutic strategy in CRC treatment.

## Methods

### Gene expression datasets retrieval

Microarray data of five gene expression projects for normal colon and rectum samples were retrieved from the Gene Expression Omnibus database (GEO), searching against query words such as colorectal cancer and CRC, on 27th September 2021 [16]. The study’s criteria for selecting samples were Homo sapiens-derived samples, excluding cell line experiments (Table 1). In the GSE41657 dataset, there were 12 normal cells, 25 carcinoma cells, and 51 dysplastic cells. Since dysplastic cells are not true carcinoma cells, we only included the normal and carcinoma cell samples in our study.

**Table 1:** Characteristics of the datasets

Dataset	Total samples	Selected samples	Platform	Reference
GSE25071	50	Normal = 4 Carcinoma = 46	GPL2986	[17]
GSE62321	57	Normal = 18 Carcinoma = 39	GPL97 [HG-U133B]	[18]
GSE8671	64	Normal = 32 Carcinoma = 32	GPL570 [HG-U133_Plus_2]	[19]
GSE41657	88	Normal = 12 Tumor = 25	GPL6480	[20]
GSE39582	585	Normal = 19 Carcinoma = 566	GPL570 [HG-U133_Plus_2]	[21]

### Identification of differentially expressed genes (DEGS)

We used the limma R package (Ritchie et al. 2015) and the GEO2R tool to normalize and identify differentially expressed genes in each dataset [22]. DEGs were selected in all 5 datasets at log fold change (log FC) = 1 or − 1 and *P* values > 0.05 as the cut-offs. Bioinfokit v2.0.1 tool in Python was used to plot volcano plots for DEGs from each dataset [23].

### Functional enrichment analysis

DEGs were arranged in descending order based on their magnitude of Log FC value after obtaining DEGs. We analyzed the top 20 DEGs from each dataset for their enrichment to understand their in-depth significance. The Supplementary Data file mentions details of the parameters submission of the top 20 DEGs. Enrichr was used to annotate the top 20 DEGs from each dataset [24]. Additionally, pathways of the top 20 DEGs were further enriched using the online tool Database for Annotation, Visualization, and Integrated Discovery (DAVID), with statistically significant pathways having a *P* value of 0.05 [25].

### Protein–protein interactions (PPI) network

STRING (Search Tool for the Retrieval of Interacting Genes) server was used to build a network of protein–protein interactions for DEGs, with the highest confidence score of 0.900 [26]. Network visualization was carried out using Cytoscape, version 3.6.1 [27].

### Cancer pathway analysis

A further analysis of the enriched pathways and their respective genes in CRC was conducted using KEGG database, an online resource integrating 18 databases categorized into systems, genomic, chemical and health information [28]. The pathways involved in cancer were examined with particular reference to the highly upregulated genes found among the DEGs of the five datasets.

### Gene expression visualization between tumor and normal tissues

Gene Expression Display Server-GEDS was used to determine gene expression levels in both tumors and normal tissues after choosing frizzled receptors (FZD) as potential targets [29]. Both TCGA and Microarray DEGs were analyzed for frizzled receptors and their expression levels. Gene expression levels of a frizzled receptor with Log FC > 1 and *P* value 0.05 were selected as possible targets [30].

### Gene expression validation between CRC and normal colorectal tissues

The Cancer Genome Atlas-TCGA (TCGA) database was examined for the purpose of validating and improving the reliability of our results [30]. TCGAbiolinks, a Bioconductor R package, was used to search, download, and prepare data for validation [31]. Using Bioconductor R package edgeR, DEGs were identified [32]. The cut-off criterion for statistically significant filtered DEGs was Log2 FC <  − 1 or Log2FC > 1 with a *P* value < 0.05.

### Prediction and enrichment of the target microRNAs

DIANA-microT-CDS was used to predict the target miRNAs as well as their functional analysis for the identified frizzled receptor [33]. All possible targets for the identified frizzled receptor were identified and downloaded, with a threshold of 0.7. Further, MicroRNA ENrichment TURned NETwork–MIENTURNET and TargetScan were also used for miRNA-target enrichment analysis to identify the potential target of the selected frizzled receptor and their network-based analysis [34, 35].

## Results

**Fig. 1:**
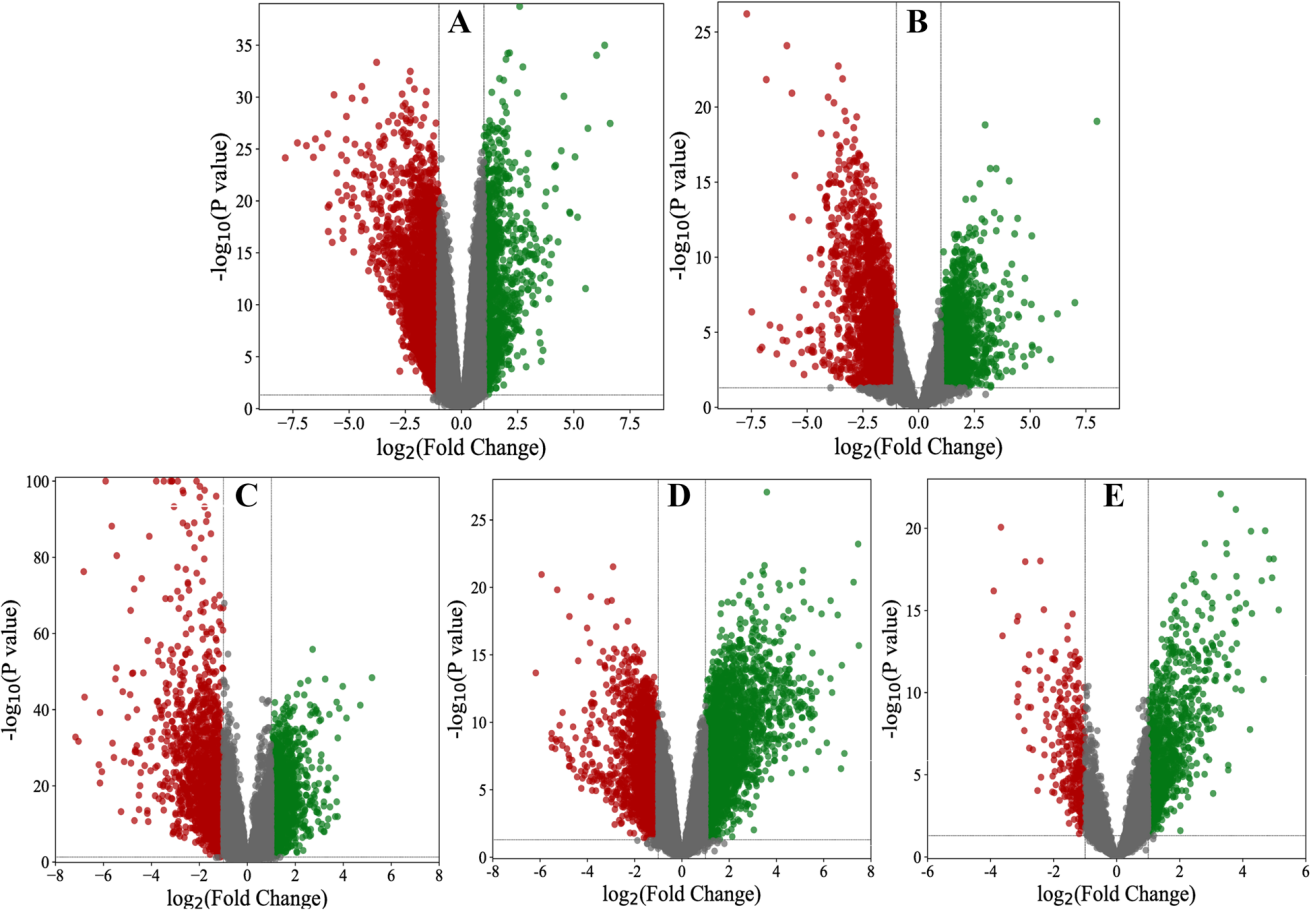
Volcano plots of DEGs. The plots show differential gene expression among the 5 datasets of this study, where red = downregulated genes, grey = normal genes, and green = upregulated genes. (**A** GSE8671, **B** GSE25071, **C** GSE39582, **D** GSE41657, **E** GSE62321)

A total of 14,227 DEGs were obtained from 5 datasets after separately analyzing the microarray datasets (Table 2 and Fig. 1). Overall, differential gene expression was more prevalent in down-regulated genes than in up-regulated genes. For further analysis, the top 20 genes from each dataset were selected from the list of overexpressed genes (Table 3).

**Table 2:** Number of DEGs from each dataset in this study

Dataset	No. of upregulated genes	No. of downregulated genes
GSE62321	427	999
GSE41657	1706	2150
GSE39582	1284	1204
GSE25071	1749	1323
GSE8671	1012	2419

**Table 3:** The top 20 DEGs that were upregulated and downregulated in five datasets

DEGs	Genes symbols				
	GSE62321	GSE41657	GSE39582	GSE25071	GSE8671
Upregulated	INHBA, FOXQ1, ASCL2, ZFAS1, CRNDE, PABPC1L, CTHRC1, CDCA5, ADAMTS2, CLDN2, CRNDE, BLACAT1, CLDN1, PSAT1, SLC6A6, EPHX4, SFTA2, DACH1, FAM3B, GOLT1A	MMP7, KRT80, PPP6R1, IRX5, CABP7, LRRC2, CLDN1, RPA4, CD86, KLK10, MMP3, ATXN3L, CEMIP, RBM22, CA9, FABP6, CGB1, FOXQ1, REG1A, FAM193B	CDH3, MMP7, MMP3, REG1A, DPEP1, SPP1, KLK10, INHBA, TACSTD2, UBD, KRT23, S100A8, CXCL8, SFRP4, S100A9, CXCL10, CLDN2, LY6G6D, SAA1, LY6E	CDH3, MMP7, MMP3, REG1A, DPEP1, SPP1, KLK10, INHBA, TACSTD2, UBD, KRT23, S100A8, CXCL8, SFRP4, S100A9, CXCL10, CLDN2, LY6G6D, SAA1, LY6E	TCN1, PINB5, CEMIP, MMP7, DEFA6, KLK10, C2CD4A, CO1B3, DPEP1, FOXQ1, MMP3, CLDN2, S100A2, MSX2, MSX2, ASCL2, CHI3L1, CRNDE, CDH3
Downregulated	IL6R, CDKN2B, DHRS9, PKIB, SLC26A2, OGN, PGM5, SORBS2, MFSD4A, MYH11, ZBTB7C, LYPD8, TRPM6, GCNT2, SCARA5, SLC26A2, ST6GALNAC6, SYNPO2, B3GALT5, SLC51B	GUCA2A, IGLJ3, IGKC, IGLV6, IGLL1, IGLJ3, LYPD8, STMN2, ADAMDEC1, ZG16, CD177, GUCA2B, IGHA2, CLDN8, CLCA1, B3GALT5-AS1, ITLN1, INSL5, GCG, CA1	SLC26A2, TCAF2, ZG16, EYA2, CLCA1, ZFP69B, ADH1C, AAAS, SLC26A3, TRIM74, KCTD17, GUCA2A, CD177, CA2, MS4A12, AQP8, LYVE1, CLCA4, CA1, CDX4	SLC26A2, TCAF2, ZG16, EYA2, CLCA1, ZFP69B, ADH1C, AAAS, SLC26A3, TRIM74, KCTD17, GUCA2A, CD177, CA2, MS4A12, AQP8, LYVE1, CLCA4, CA1, CDX4	PYY, TRPM6, CLCA4, CD177, CXCL13, PTL1, TPH1, CA4, CHGA, INSL5, S4A12, CLDN8, GUCA1, TPH1, GUCA2A, AQP8, SST, PYY, CA2B, GCG

### The Wnt signaling pathway is highly enriched in CRC patients

**Fig. 2:**
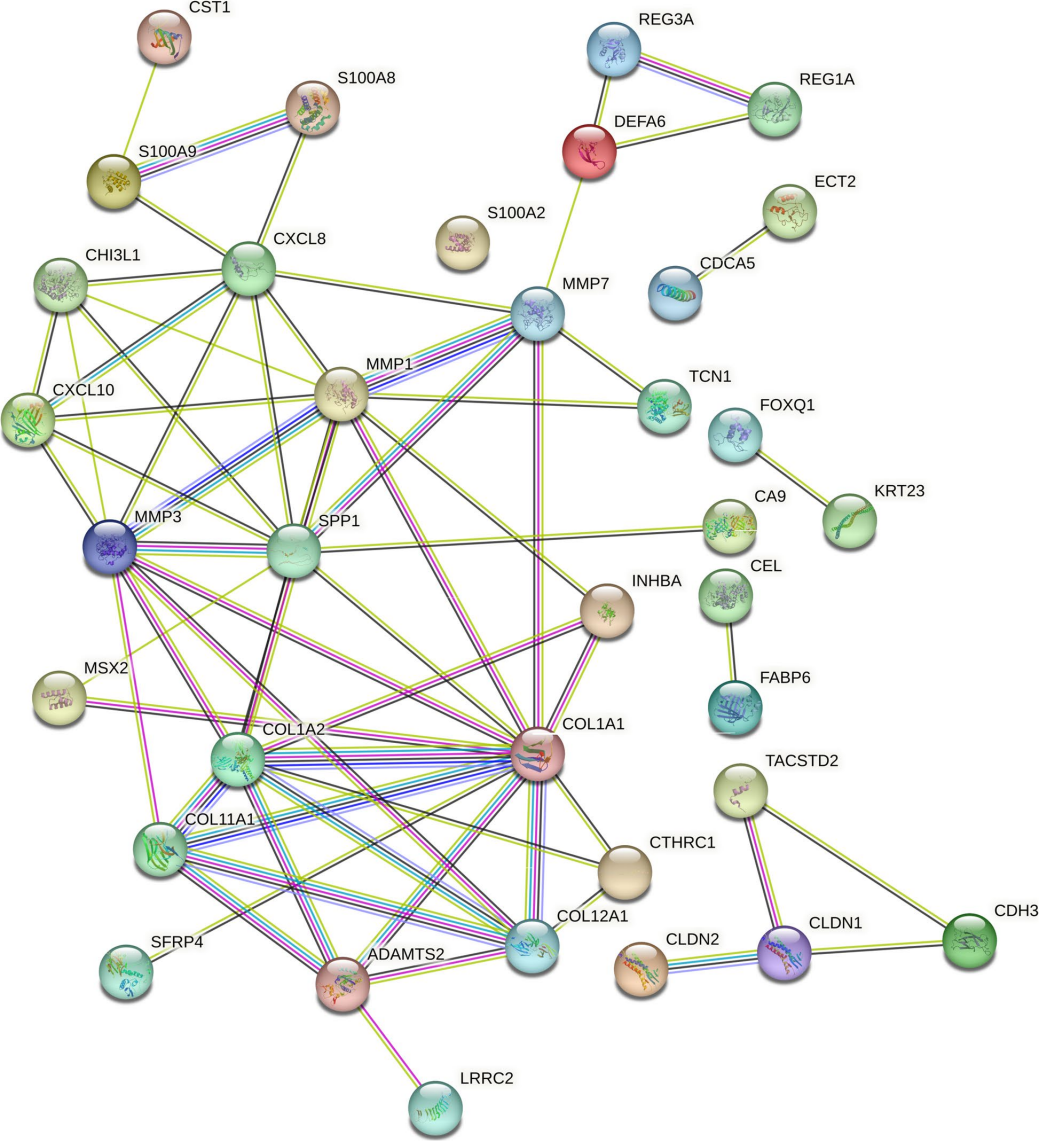
The protein–protein interaction network. The network depicts interactions among the top up-regulated DEGs, excluding disconnected nodes in the network. (Color nodes—query proteins or the first shell of interactors, white nodes—second shell of interactors, filled nodes—proteins with known or predicted 3D structures, empty nodes—proteins with unknown 3D structures)

The enrichment analysis of the top 20 DEGs from each dataset demonstrated that these genes were involved in 16 different pathways, taking the cut-off criterion for statistically significant pathways being *P* value < 0.05. Although a number of pathways related to cancer, such as PI3k-Akt, HIF-1 among others, were all enriched among the top DEGs in these datasets, Wnt signalling was significantly enriched compared to other pathways, with genes such as SFRP4, MMP7, among others, being involved. The protein–protein interaction network revealed that the Wnt signalling pathway genes from the five datasets were among the genes with the highest number of interactions, for example, MMP7 with 7 interactions and MMP3 with 9 interactions, when combined together and visualized (Fig. 2). Considering the higher levels of enrichment for Wnt pathway genes and their interactions, we hypothesized that inhibition of the pathway could suppress colorectal cancer pathogenesis.

**Fig. 3:**
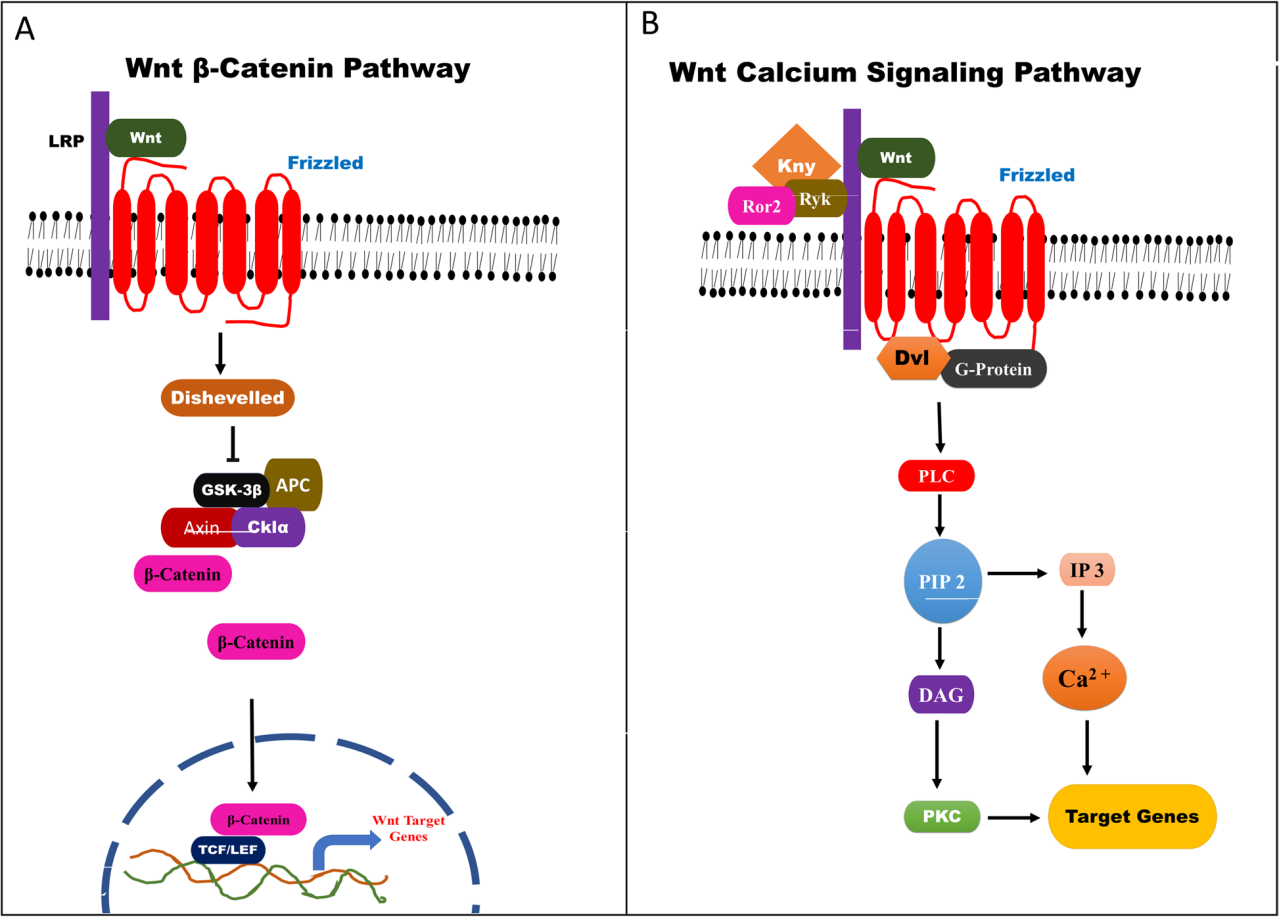
The Wnt signaling pathway. Canonical Wnt pathways are depicted in **A**, while non-canonical Wnt pathways are depicted in **B**

Further analysis of the Wnt signalling pathway enrichment in CRC was conducted using KEGG Pathways and indicated the pathway serves as a gateway to several key genes and pathways in CRC; some of which were identified among DEGs that were overexpressed in all five datasets (Table 3). In this pathway, the Wnt ligand binds to frizzled family receptors or ROR1/ROR2 and RYK family receptors, stimulating signalling cascades, either canonical or non-canonical, that result in transcription of the Wnt target genes (Fig. 3). In light of this fact, we narrowed down our hypothesis that inhibiting the expression of FZD receptors would suppress the binding of Wnt ligands and, as a result, inhibit this highly implicated signaling pathway.

### Pathway and functional analysis of FZD receptors

**Fig. 4:**
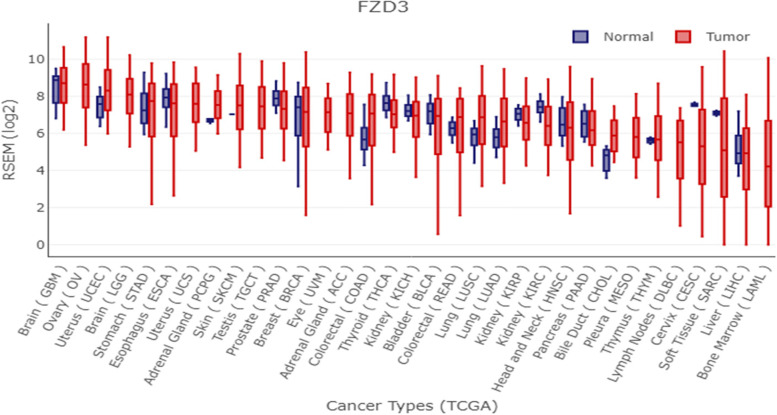
Expression levels of FZD3 receptor in different cancer types. Colorectal cancer is one of such cancer types in which the expression of FZD3 in normal is more than doubled in tumor tissues

**Fig. 5:**
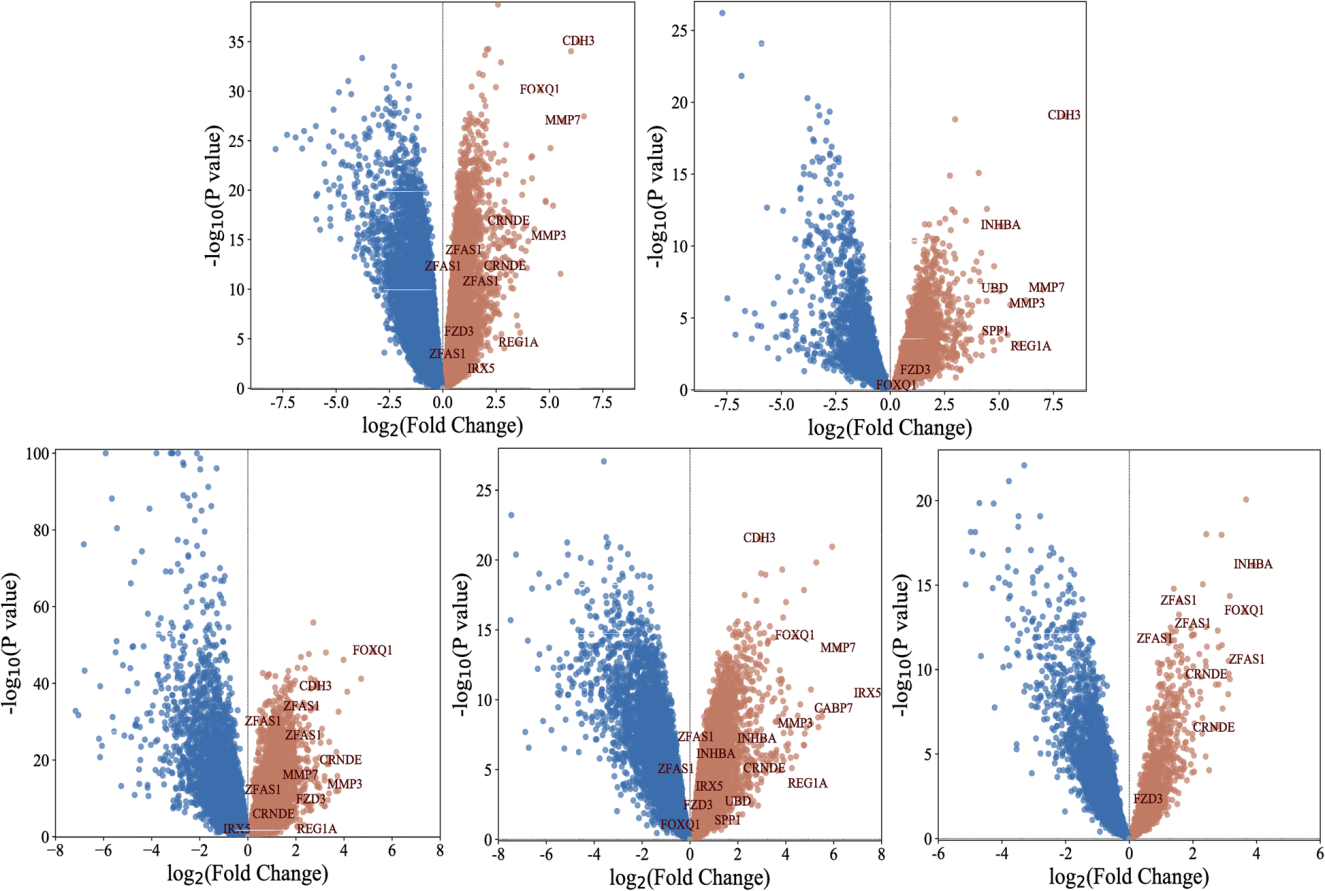
The volcano plots show the differential expression of FZD3 across our 5 datasets combined with the GEDS dataset. FZD3 was shown to be upregulated together with other genes in normal colorectal cells compared to cancerous cells. Blue = downregulated, grey = normal regulation, brown = upregulated genes. (**A** GSE8671, **B** GSE25071, **C** GSE39582, **D** GSE41657, **E** GSE62321)

**Fig. 6:**
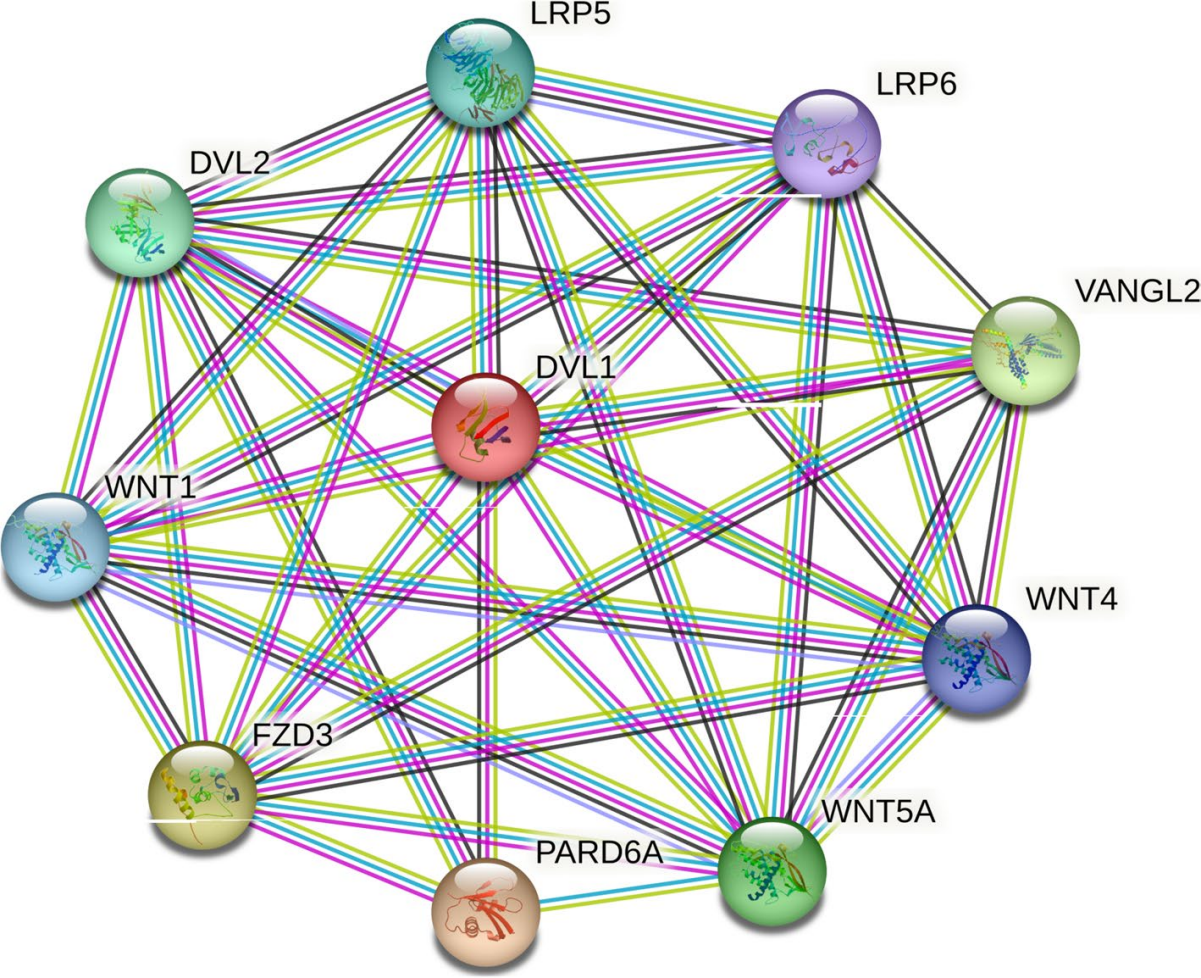
Protein–protein interaction network of FZD3. FZD3 interacts with other Wnt signaling pathway genes in the network. The inhibition of this gene can affect the entire pathway, inhibiting colorectal cancer progression. (Color nodes—query proteins or the first shell of interactors, white nodes—second shell of interactors, filled nodes—proteins with known or predicted 3D structures, empty nodes—proteins with unknown 3D structures)

To test our hypothesis of inhibiting FZD receptors, the expression levels of each of the 10 FZD receptor family members were compared between normal and cancerous colorectal tissues using the Gene Expression Display Server (GEDS) and found that FZD receptors have higher expression levels in cancerous tissues than normal tissues, with some receptors having a higher expression level in CRC than others such as FZD3 (Fig. 4). In addition to GEDS database, we further analyzed the expression levels of all FZD family receptors in our study dataset and found that FZD3 receptor was up-regulated across all 5 datasets, unlike other receptors. To validate this result, we further performed TCGA analysis with TCGA-COAD and TCGA-READ datasets and found FZD3 gene was up-regulated in tumor tissues compared to normal ones, with a Log FC that correlates with the result of our datasets (Fig. 5). Therefore, downregulating the FD3 receptor would inhibit the Wnt signaling, thereby suppressing tumor proliferation and progression in CRC since the interaction between the receptor and other proteins will be affected (Fig. 6).

### Inhibition of FZD3 receptor using miRNAs

**Fig. 7:**
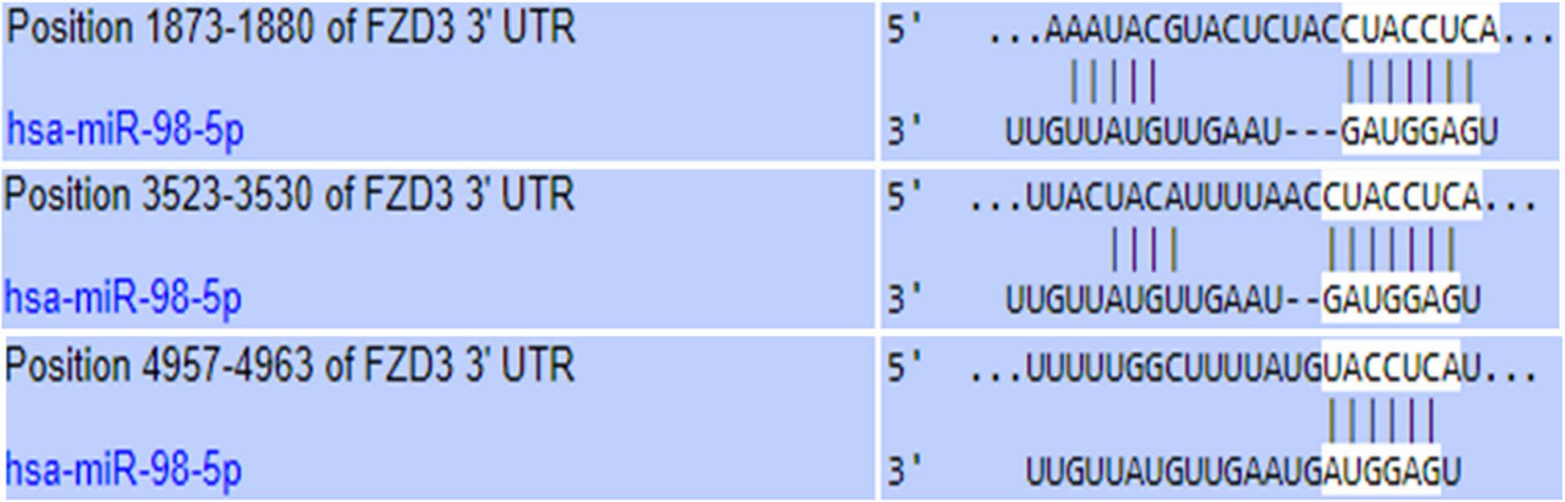
FZD3 and miRNA binding. miR-98-5p is depicted as a putative inhibitor of FZD3 via its binding to the 3'-UTR of FZD3

Several studies have identified and reported a number of microRNAs (miRNA) as potential frizzled receptor inhibitors in CRCs as well as other cancer types when miRNAs bind to the 3′ untranslated regions (3′UTRs) of target FZD mRNAs and inhibit their post-transcriptional expression. Having identified FZD3 as a candidate receptor to inhibit Wnt signaling, further investigations were made to identify potential miRNAs which targeted the 3′UTR of FZD3 mRNA using computer-based algorithms. Using DIANA-microT-CDS, 606 Homo sapiens microRNAs (hsa-miRNAs) with a threshold of 0.7 were identified as potential FZD3 targets. The miRTarBase of MIENTURNET web tool provided us with five miRNAs as potential targets for the FZD3 receptor after further analysis of the identified potential targets. The miRNAs include hsa-miR-7856-5p, hsa-miR-3658, hsa-miR-31-5p, hsa-miR-98-5p, and hsa-miR-3653-3p. A functional enrichment analysis conducted using the MIENTURNE web tool found that only 2 of the 5 miRNAs examined were enriched in the Wnt signaling pathway. These two miRNAs are hsa-miR-31-5p and hsa-miR-98-5p. Then, based on recently published literature, the two microRNAs were further studied and Hsa-miR-98-5p was chosen as the best target for the FZD3 receptor on the grounds that recent research has indicated hsa-miR-31-5p having oncogenic properties in CRC [36]. Finally, TargetScan analysis revealed that hsa-miR-98-5p binds three different positions on FZD3 mRNA: 1873, 3523, and 4957 (Fig. 7).

## Discussion

Colorectal cancer incidences and mortality rates have decreased since the mid-1980s, but this malignancy accounted for more than 1.5 million new cases worldwide and nearly 1 million cancer-related deaths in 2020. Despite the fact that CRC burdens vary across countries, increasing incidences are reported in countries with high Human Development Indexes. In addition, CRC continues to be a big problem in developing countries whose health care systems are still poor, and CRC treatments are still unaffordable. Therefore, it is imperative that treatment approaches be improved to alleviate this deadly condition and improve survival rates. Routine CRC treatment strategies such as chemotherapy, immunotherapy, radiotherapy, and surgery have saved so many lives however, targeted therapy due to advancements in health science brings more specificity and increases survival among CRC patients.

Targeted therapy in CRC aims to block different critical pathways that are responsible for cell growth and proliferation, angiogenesis, migration, differentiation, and anti-apoptosis by using small molecules such as monoclonal antibodies and miRNAs. These molecules penetrate cells and inhibit the target pathway, interfering with the growth of tumors and, in some cases, causing apoptosis [37]. Pathways that offer potential sites for targeted therapy in CRC include among others; Wnt/β-catenin, HGF/c-MET pathway, notch, hedgehog, and EGFR-related pathways [37]. In spite of being referred to as non-coding RNA molecules, miRNAs have been shown by a number of studies to play an important role in regulating 60% of human genes post-transcriptionally and in being associated with cancer [38]. Hence, we sought to inhibit FZD3, one of the frequently up-regulated frizzled receptors of the Wnt signaling pathway in CRC, by using miR-98-5p, a rarely reported miRNA in this disease.

**Fig. 8:**
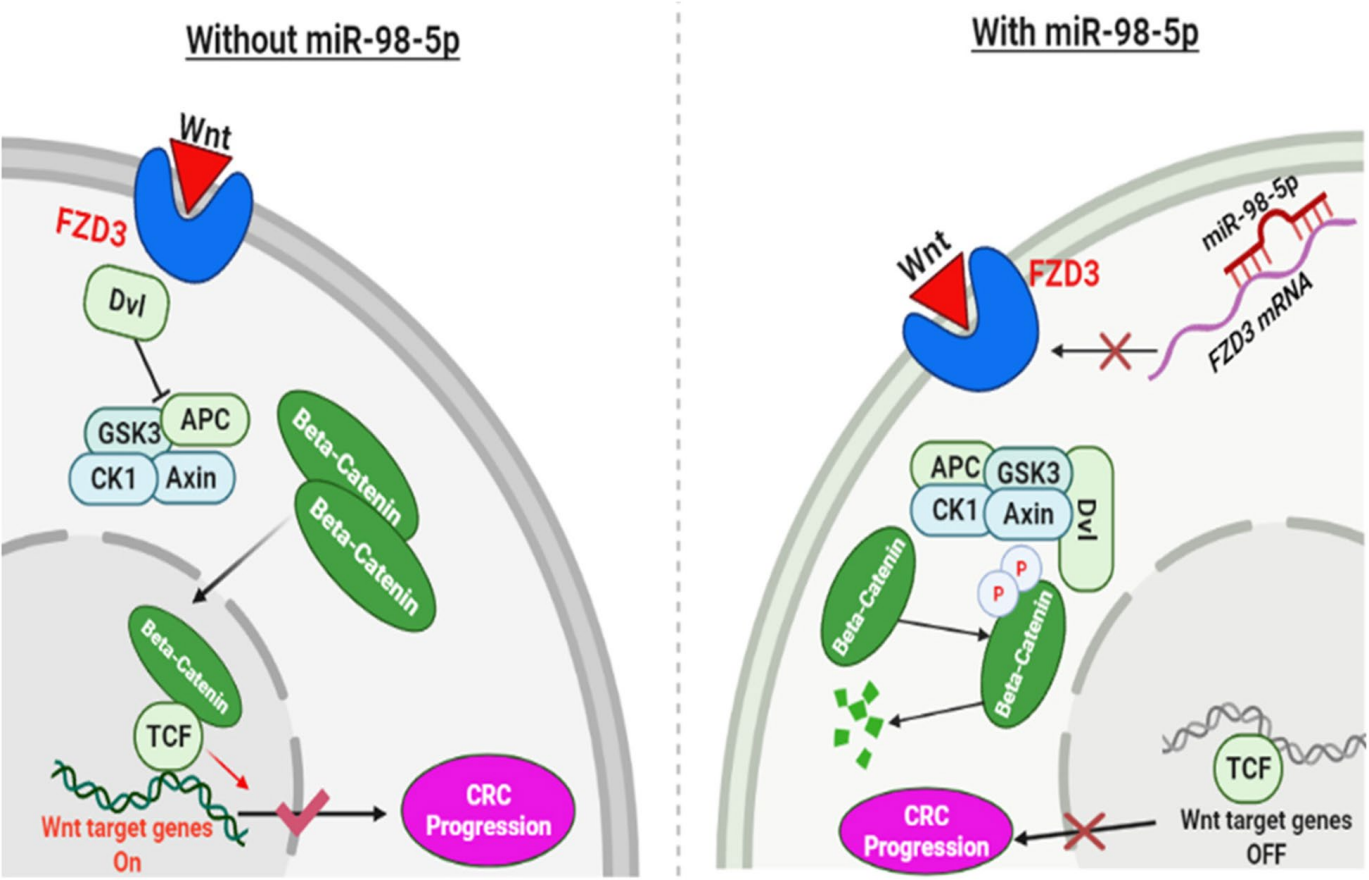
Inhibition of CRC progression*.* The illustration depicts the putative progression of Wnt signaling pathway with/out miR-98-5p in CRC cells

FZD3 receptor expression was determined using differential gene expression analysis on five datasets and found that the change in levels of expression between tumor and normal samples was significant enough for it to be a DEG alongside other genes. Following an analysis of gene ontology enrichment, it was found that FZD3 was significantly enriched in the Wnt signaling pathway along with many other genes within the pathway, which correlates with recent studies which implicate Wnt signaling in the development of CRC [9, 39, 40]. Further analysis of the GEDS web server datasets revealed that FZD3 expression was higher in CRCs, which validated our findings. Furthermore, we have constructed a PPI and found FZD3 receptor interacting with key WNT signaling pathway genes, including DVL, WNT1, WNT5, LRP6, and VANGL2 that were also found to be upregulated in our study data. It was also found that sFPR1 (secreted frizzled receptor protein 1), a Wnt antagonist, was downregulated in all the datasets, which was previously reported to be down-regulated by the up-regulation of FZD3 [41]. Finally, it has been reported in several studies that FZD3 expression is correlated with Wnt target genes, Cyclin D1 and c-My, which we also found to be true in our analysis [14]. Therefore, on the basis of these results and literature references, it seems reasonable to suggest that FZD3 plays a crucial role in the Wnt pathway and that its inhibition would inhibit the pathway (Fig. 8).

Recent studies have demonstrated that microRNA miR-98-5p inhibits tumor proliferation, migration and invasion by targeting the Wnt signaling pathway-related genes in various cancers including ovarian cancer [42], glioblastoma [43], gastric cancer [44] in non-small cell lung cancer [45], and pancreatic ductal adenocarcinoma [46]. The findings represent miR-98-5p as a potential target of FZD3, one of the major receptors of the Wnt pathway. Computational analysis showed that the FZD3 mRNA contained the binding sites for miR-98-5p in its 3′-UTR, which is a key feature in the miRNA post-translational gene regulation mechanism. However, miRNA prediction algorithms can barely confirm that miR-98-5p targets FZD3 directly in CRC samples. To validate the study results, luciferase reporter assays should be done to compare the behavior of a wild-type (WT) as well as the mutated (MUT) FZD3 in the 3’UTR binding site. The difference in fluorescence between FZD3-WT and FZD3-MUT will confirm that miR-98-5p is directly targeting the FZD3 gene. FZD3 and miR-98-5p could be forming an axis that inhibits Wnt signaling and CRC in general; however, the involvement of other target genes in the process cannot be ruled out. It is important that all the predicted target genes by at least two miRNA prediction algorithms are enriched in Wnt pathways by gene ontology and KEGG in order to validate the mechanism by which miR-98-5p inhibits Wnt signaling pathways. The mRNA expression levels of such genes can then be measured with miR-98-5p mimic and inhibitor, respectively.

## Conclusion

In conclusion, this study demonstrated that FZD3 is upregulated in CRC along with other crucial genes of the Wnt signaling pathway. Moreover, this provides evidence that miR-98-5p may inhibit the expression of FZD3, which may lead to reduced proliferation and metastasis of colorectal cancer cells, and these findings can be used in the development of target-based therapies for CRC patients. It is essential, however, that these findings be validated by basic research in the future to determine the mechanism by which miR-98-5p regulates CRC cells, both in vivo and in vitro.

## Supplementary Information

**Additional file 1.** Supplementary Data.

## Data Availability

Datasets used in this study are available in public databases as indicated in the paper citations. However, generated data and figures during this study are available on request to the corresponding author.

## Abbreviations

CRC: Colorectal cancer
miRNA: MicroRNA
FZD: Frizzled receptor
GEO: Expression Omnibus database
DEGs: Differentially expressed genes
KEGG: Kyoto Encyclopedia of Genes and Genomes
WNT: Wingless-related integration site
